# Co-remediation of Pb Contaminated Soils by Heat Modified Sawdust and *Festuca arundinacea*

**DOI:** 10.1038/s41598-020-61668-x

**Published:** 2020-03-13

**Authors:** Yan Zhang, Xuemei Wang, Hongbing Ji

**Affiliations:** 1 Beijing Key Laboratory of Resource-oriented Treatment of Industrial Pollution, School of Energy and Environmental Engineering, University of Science and Technology Beijing, Beijing, 100083 China; 2 Beijing Municipal Key Laboratory of Resource Environment and GIS, College of Resource Environment and Tourism, Capital Normal University, Beijing, 100048 China

**Keywords:** Environmental sciences, Environmental chemistry, Environmental impact

## Abstract

This research aimed to explore the potential and mechanism of heat modified sawdust combined with *Festuca arundinacea* for the remediation of Pb-contaminated soil. We determined Pb concentration and biochemical indices in plants and soils, analyzed microbial communities in soil, and studied Pb distribution in subcellular and tissues. Under co-remediation of 5% material addition and *Festuca arundinacea*, the concentration of Pb in soil decreased. Pb toxicity of *Festuca arundinacea* was alleviated by 2% material addition through the promotion of plant growth and reduction of oxidative stress. In addition, soil enzyme activities and microbial community in contaminated soil were promoted by the application of co-remediation. *Festuca arundinacea* cell wall accumulated a large amount of Pb, and the addition of material promoted the accumulation of Pb in *Festuca arundinacea* root. The concentration of Pb in the shoot of the plant treated with 2% material was higher than that of the plant treated with 5% material, and the damage of *Festuca arundinacea* leaves was lower under 2% treatment. The combination of heat modified sawdust and *Festuca arundinacea* promoted the adsorption of Pb by plants, and protected the growth of plants.

## Introduction

Pb pollution has become increasingly prominent in recent years, with the gradual development of society. Soil is the basic growth environment for food and cash crops, and pollution cannot be ignored. According to the results of the national soil pollution survey released by the Chinese government in 2014, the soil environmental quality of cultivated land in China is not optimistic, and the soil problem of industrial and mining wasteland is prominent^1^. The over-standard rate of Pb contaminated soil in China is 1.5%^2^. Duan *et al*.^3^ investigated the Pb concentration of 2,489 sampling points in China and found that the current pollution level of Pb in soil is severe, thereby posing a serious threat to the health and ecological stability of Chinese residents. Everyone hopes that the soil problem of Pb pollution that is closely related to human life can be truly solved. Many scholars used physical, chemical or biological technology to reduce the total or available content of Pb in soil to decrease toxicity and accumulation of Pb in crops^4^.

Research on recycling and reuse of biomass wastes is a very important strategy to improve the utilization of bio-energy resources^5^. Heat modified biomass materials can effectively stabilize heavy metals in soil, thereby alleviating the heavy metal toxicity in the environment^6^. X.D. Song *et al*.^7^ showed that the leaching of heavy metals can be prevented by applying biochar to the soil. Moreover, the accumulation of heavy metals in garlic can be inhibited, and the yield and quality of garlic can be improved. The application research of sawdust, ay-product of industry and agriculture, has been the focus of an increasing number of studies by environmental protection researchers. Sawdust has a substantial effect on adsorption and removal of dyes, heavy metals, oil and other pollutants. Memon *et al*.^8^ used cedar sawdust discarded in local furniture market to adsorb toxic metal Cd in aqueous solution, and the results showed that cedar sawdust was an economic type of Cd^2+^ adsorbent. Avelino Núñez-Delgado^9^ studied the adsorption and desorption ability of pine sawdust and oak ash to Cr (VI), and these two materials showed high application value in removing Cr from polluted soil and had great potential as biosorbents for Cr.

Phytoremediation is widely used in the treatment of soil pollution because of thoroughness and environmental friendliness. Herbaceous plants have the characteristics of large biomass, fast growth, and strong stress resistance^10^. Studies have shown that herbaceous plants have important value in the field of phytoremediation of heavy metal contaminated soil, and several species have good tolerance to heavy metal ions^11^. *Festuca arundinacea* is a kind of herbaceous plant with high utilization rate in China. It is suitable for growing in different soil and environmental conditions, with a wide range of growth, especially in Yunnan, Guizhou, and other areas in South China. Previous studies found that *Festuca arundinacea* can tolerate 200 mg/kg of Cd in soil without any toxic symptoms^12^. *Festuca arundinacea* is most commonly used for the plant stabilization of Cu, Zn and Pb in polluted soil in European countries^13^. However, using *Festuca arundinacea* for super enrichment have some shortcomings, such as slow growth rate and low biomass. They may have strong regional distribution and are at a competitive disadvantage when transplanted to other places. One strategy is to use physics, chemistry, biology, agronomy, and other technologies to enhance the absorption capacity of hyperaccumulators or locally dominant plants.

Chemical-phytoremediation technology can be used as a safe, economical and long-term method to eliminate heavy metals in soil^14,15^. However, the combination of chemical passivation and plant extraction may have unexpected effects. On the one hand, applying heat modified biomass to soil can reduce the bioavailability of metals^16,17^. On the other hand, the application of materials will enhance extraction potential of plants, thereby resulting in enhanced plant activation of heavy metals. The two processes have seemingly contradictory effects. Paz-Ferreiro *et al*.^18^ reviewed the potential of combining plant and biochar for remediation of contaminated soils, and suggested that practical research was needed to determine the sustainability of the co-remediation technology. Therefore, we applied heat modified sawdust (HMS) in Pb-contaminated soil and combined it with phytoremediation of *Festuca arundinacea* to study the co-remediation potential and effects of Pb-contaminated soil. The effects of co-remediation on the behavior of Pb in soil were studied through the determination of Pb content and fractions in soil. The effects of co-remediation on soil environment were analyzed by measuring soil organic matter content, enzyme activity and microbial community. The effects of co-remediation on phytotoxicity of *Festuca arundinacea* were studied by measuring its enzyme activities. The effect of co-remediation on Pb accumulation in *Festuca arundinacea* was studied by measuring the distribution of Pb in its organs and subcells.

## Materials and Methods

### Experimental soil, remediation material, and plant

Pot soil (0–20 cm) used for the experiment was collected from Maomaochang Pb/Zn mining area, Hezhang County, Guizhou Province, China (26°57′30″N, 104°29′40″E). Soil near the sampling point has been seriously polluted by Pb. Impurities such as large stones in the collected soil were screened out. The soil was air-dried, ground, and pass through a 10-mesh sieve. Part of the treated soil was used for cultivation experiments, and the others were ground again to pass 200 mesh sieves to analyze the physicochemical properties. Physicochemical properties of the soil were determined according to the analytical methods described by Ji^19^. Table S1 shows the basic physicochemical properties of pot experiment soil.

Soil remediation material used in the study was a mixture of sawdust ash (SA) and sawdust biochar (SB) (SA:SB w/w = 1:2). SA is the uncontrollable particulate matter produced by aerobic combustion of sawdust at 400 °C–700 °C, which mainly includes ash and unburned carbon in fly ash. The unburned carbon content in fly ash from wood combustion is higher, about 7–40%, thereby increasing the emission and carbon content of SA (26.63%)^20^. SB is the pyrolyzed product of sawdust, which was heated up to 500 °C at a slow rate and held for 2 h under constant N_2_ gas protection. The material was ground to pass a 1 mm sieve before use. Physiochemical characteristics of SA and SB are presented in Table S2.

The seeds of *Festuca arundinacea* were purchased from Beijing Guoren Horticulture Co., Ltd.

### Experimental design

Each experimental pot contained 1.5 kg contaminated soil. According to the demand of plant growth, nutrient solution was sprayed evenly in the soil ensure the nutrient supply of plants^21^. A week later, remediation materials were added in the soil according to the treatments, and were evenly mixed with the soil. In our previous paper, we studied the effect of different dosage materials on the remediation of Pb pollution^22^. According to the previous research results, combined with the research of other scholars^23^, we have determined that the proportion of materials added in this study is 2% and 5%. The treatments in this study were: pot soil without *Festuca arundinacea* planting and no material addition served as the control (CK), *Festuca arundinacea* planting and no material addition (FA), 2% material addition (w/w) without *Festuca arundinacea* planting (2% NP), *Festuca arundinacea* planting +2% material addition (w/w) (2% FA), 5% material addition (w/w) without *Festuca arundinacea* planting (5% NP), and *Festuca arundinacea* planting +5% material addition (w/w) (5% FA). Each treatment was tested in triplicates. The experimental treatments were shown in Table S3. The soil was balanced for one week, and deionized water was added to the soil during the period to keep the soil moisture content at 60% of the field capacity. The *Festuca arundinacea* seeds were spread in the soil and cultivated for 60 days. The soil and plant were treated according to the method described by Zhang^21^ and Ji^19^.The tender branches and roots of the harvested plants were washed with deionized water to remove soil and dirt^21^. Meanwhile, the rhizosphere soil samples of the plants were collected. One part of the plant and soil was used to determine the content of Pb, whereas another part was used for the analysis of physiological indices^19,21^. A portion of the soil sample was cryopreserved in a 10 mL sterile centrifuge tube for sequencing and microorganism test^19^.

### Sample determination and analysis

#### Pb content and fractions in soil

The treatment and determination of soil samples were carried out according to our previous experimental steps^21^, and the specific methods are listed in Supplementary Material.

The standardized procedures i.e., synthetic precipitation leaching procedure (SPLP), diethylenetriaminepentaacetic acid (DTPA) extraction method, and European Community Bureau of Reference (BCR) sequential extraction procedure were used for toxicity characterization and evaluation of potential risk of Pb in terms of metal leachability and bioavailability.

SPLP was used to simulate the exposure and migration characteristics of wastes under acid rain, i.e., air pollution caused by heavy industry and coal combustion^24^. It has been widely applied in evaluating the remediation effect of contaminated soils^25^. The bioavailability of Pb in the soils was assessed by DTPA extraction method^26^. Pb fractions in soil were determined using BCR method developed by the Standard Measurement and Testing Program of the European Community^27^. On the basis of synthesizing the existing methods for extracting heavy metals from sediments, the European Community Bureau of Reference proposed a three-step extraction method (BCR method) for speciation analysis of heavy metals in soil and sediment samples in 1993^28,29^. The experimental methods of SPLP, DTPA extraction method, BCR method and the specific meaning of each fraction extracted by BCR method are described in the Supplementary Material. The experimental methods of speciation extraction and toxic leaching of Pb in soil are shown in Table S4.

#### Pb content in *Festuca arundinacea*

The total amount of Pb in *Festuca arundinacea* was determined according to our previous experiment step^21^, and the determination of Pb content in subcellular of *Festuca arundinacea* was carried out according to the method reported by Zhang *et al*.^30^. The specific experiment steps are listed in Supplementary Material.

#### Distribution of Pb in plant tissues

The distribution of Pb in fresh root or shoot samples of *Festuca arundinacea* was analyzed by fluorescence labeling. Leadmium Green AM stock solution was made by adding 50 µL DMSO to one vial of dye. The solution was mixed well and protected from light. Leadmium Green AM dye working solution was prepared by diluting Leadmium Green AM stock solution in saline (v:v = 1:10). The plant samples were cut into 40 μm thickness by a freezing slicer and were placed at room temperature for 30 min. The samples were washed thrice with 1 × PBS and dyed with the proper amount of diluted Pb dye solution. The samples were incubated in a wet box for 1 h, washed thrice with 1 × PBS and sealed. The samples were observed by laser scanning confocal microscopy.

#### Assays of physiological indexes

The rhizosphere soil was air dried and ground to pass through a 100-mesh sieve. The ground samples were mixed with ultrapure water (w:v = 1:2). The samples were oscillated horizontally at room temperature for 24 h at a speed of 180 ± 20 r/min and centrifuged for 15 min at 4,000 r/min. The filtered supernatant was stored as soil DOM sample for testing. The DOC value (mg/L) was determined by total organic carbon (TOC) analyzer. The absorbance of DOM samples at 254, 260, 280, 250 and 365 nm were determined by UV-Vis spectrophotometer, which was used to indicate the macroscopic information of aromaticity and molecular weight of DOM. Specific parameters are described in Table S5.

A part of fresh plant samples was used to determine the activities of peroxidase (POD), catalase (CAT) and superoxide dismutase (SOD) and the content of malondialdehyde (MDA) and chlorophyll. Specific methods for enzyme activity determination are shown in Supplementary Material. According to the manufacturer’s instructions, an E.Z.N.A.1 soil DNA kit (Omega Bio-Tec-Inc., USA) was used to extract total DNA. The extraction process was described by previous studies^19^.

### Data processing and statistical analysis

#### Data analysis

The removal rate was calculated by measuring the content of Pb in the soil before and after planting the plants.

1 $${\rm{Removal}}\,{\rm{rate}}=({{\rm{C}}}_{before}-{{\rm{C}}}_{after})/{{\rm{C}}}_{before}\ast 100 \% $$

where C_*before*_ is the concentration of Pb in the soil before experiment and C_*after*_ is the concentration of Pb in the soil after experiment.

The phytoextraction ability of plant is evaluated by bioconcentration factor (BCF) and translocation factor (TF)^31–33^.

2 $${\rm{BCF}}={{\rm{C}}}_{plant}/{{\rm{C}}}_{Soil}$$

3 $${\rm{TF}}={{\rm{C}}}_{shoot}/{{\rm{C}}}_{root}$$

where C_*plant*_ and C_*soil*_ are the concentration of Pb in whole plant and soil after the culture experiment; C_*shoot*_ and C_*root*_ are the concentration of Pb in the shoot and root of plant after the culture experiment.

#### Statistical analysis

Standard materials were included for data quality control. The data was analyzed according to the method described by Zhang^21^. The difference and significance of Pb concentrations in soil and plant were analyzed by one-way ANOVA using SPSS Version 19.0^21^. The effects of experimental factors and the influence of treatments on soil characteristics, plant physiological parameters, and soil microbial properties were evaluated by two-way ANOVA^21^. The ANOVA was followed by post hoc Bonferroni t-tests^21^.

All curves were drawn using Origin Version 8 and Excel 2013. R language tools were used to draw microbial communities and heatmap^21^.

## Results

### Effects of co-remediation on the content and fractions of Pb

#### Concentration and removal rate of Pb

Table S6 shows the concentration and removal rate of Pb in soil after co-remediation experiments. We compared the removal rate of Pb in soil after the different treatments, and found that the removal rate of Pb after planting *Festuca arundinacea* was higher than that in soils without plants. The removal rate of Pb in the soil with 5% remediation materials was higher than that in other soils. The highest removal rate of Pb was 18.02% under 5% FA co-remediation treatment.

#### Leaching toxicity and bioavailability of Pb

**Figure 1:**
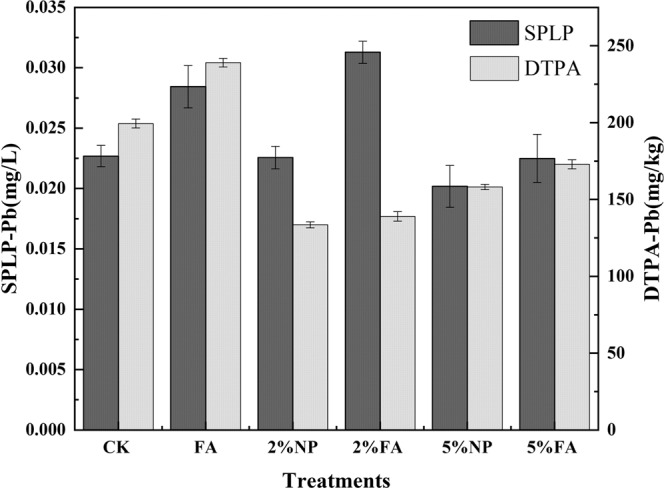
Leaching ability and bioavailability of Pb in soil samples after culture.

SPLP was applied to evaluate the soil leaching characteristics and stabilization technology under acid rain, and leachability of Pb in the treatment soils is presented in Fig. 1. Compared with CK, the leaching toxicity of Pb was reduced by remediation without *Festuca arundinacea*, and the leaching concentration of Pb was reduced by 10.59% under 5% NP treatment. The leaching concentration of Pb was increased by 2% FA co-remediation process, which was 0.0313 mg/L. The instability of Pb observed was consistent with the decrease in leaching rate, which was observed in previous studies, but this paper confirmed that phytoremediation can increase the leaching capacity of Pb. The reduction of Pb-bioavailability in co-remediation soil was evident (Fig. 1). Compared with CK, DTPA-Pb in soil after adding materials showed a decreasing trend, and the bioavailability of Pb was lowest under 2% NP treatment (133.55 mg/kg).

#### Speciation changes of Pb in soil

**Figure 2:**
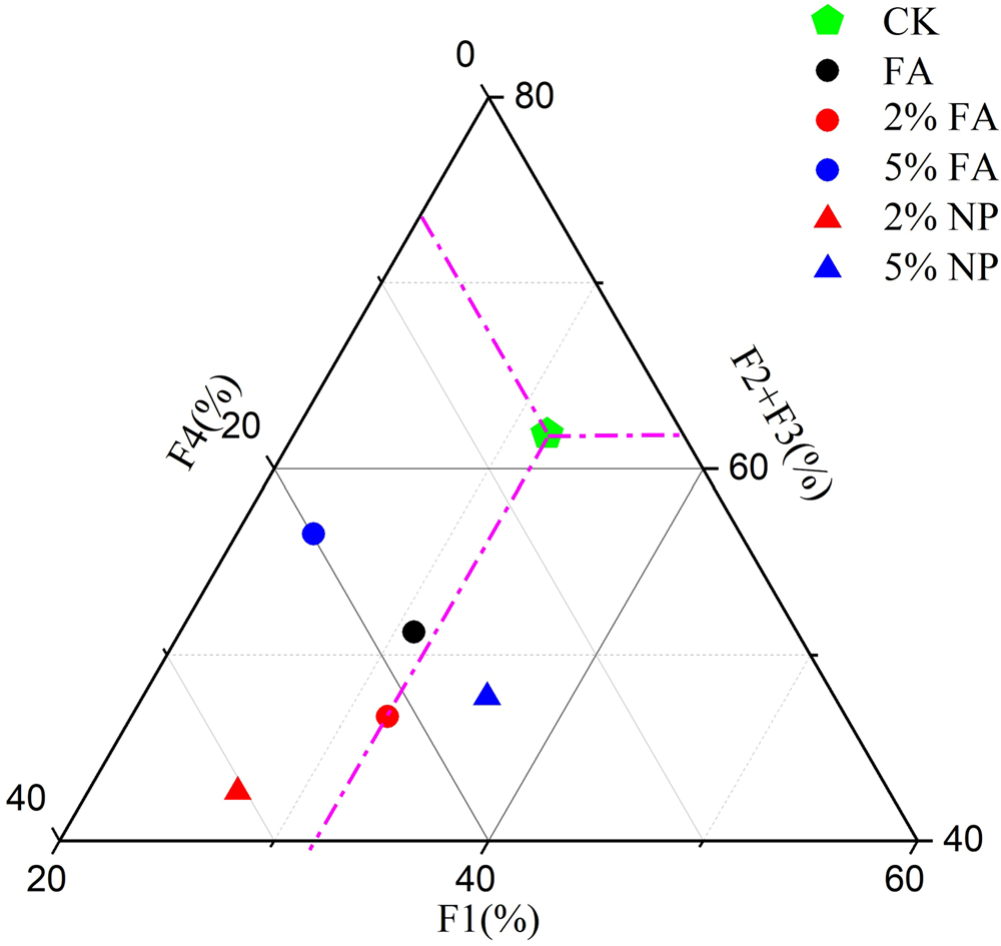
Fractions of Pb in treated and untreated soils. F1: Water-soluble and exchangeable Pb; F2: Reducible Pb bound to Fe and Mn; F3: Oxidizable Pb bound to organic matter and sulphides; F4: Residual Pb bound to silicate minerals.

Figure 2 shows the distribution of Pb fractions in the soil after remediation. Overall, the percentage of F1 in the soil decreased, and the percentage of residual Pb in the soil increased. If the decrease of F1, F2, and F3 fractions and the increase of F4 speciation were considered the best conditions for judging the remediation, 5% FA and 2% NP had better effect on soil remediation. Compared with the soil without remediation materials, the direct/potential toxicity fractions of Pb decreased after co-remediation.

We compared the four fractions in Fig. S1 in the Supplementary Material to observe the changes of Pb fractions more intuitively. As shown in Fig. S1 in Supplementary Material, the proportion Pb in F3 decreased after remediation, whereas the proportion of F2 and F4 increased. Compared with FA, the co-remediation with 5% addition can reduce the percentage of F1 fraction by 23.66% and increase the percentage of F2 fraction by 11.40%. Although the main fractions of Pb in soil varied with treatments, the co-remediation transformed the F1 fraction with strong migration and transformation behavior into a more stable residual fraction.

### Effects of co-remediation on rhizosphere soil conditions

#### DOM in rhizosphere soil

Absorption values (per unit concentration) and UV/VIS at specific wavelength, such as SUVA254, SUVA260, A280, and A250/A365 are often used to indicate macroscopic information such as aromaticity and relative molecular weight of DOM^34^. Table 1 lists UV/Vis parameters of DOM in treated soil. The content of DOM in soil varied with different remediation methods, and the co-remediation method increased the content of DOM in soil. The aromatization degree weakened, and humus transformed to non-humus in the repair process with *Festuca arundinacea* participation by analyzing the values of SUVA254 and A280. However, due to the interaction of materials and plants, no substantial correlation was observed between the dosage of materials and the content of DOM in soil. Phytoremediation changed the types of organic matter in soil. The amount of macromolecule organic matter in soil planted with *Festuca arundinacea* was less than that in soil without plants.

**Table 1:** UV/Vis parameters of DOM in treated soil.

	DOM (mg/kg)	A_250_/A_365_	SUVA_254_	SUVA_260_	A_280_
CK	283.4	20.00	2.29	2.03	0.091
FA	265.2	53.00	1.29	0.96	0.05
2% NP	332.6	27.33	1.61	1.18	0.086
2% FA	554	103.00	1.80	1.05	0.085
5% NP	251	43.00	2.02	1.38	0.083
5% FA	358.8	21.83	1.40	1.14	0.067

#### Microorganisms in rhizosphere soil

**Figure 3:**
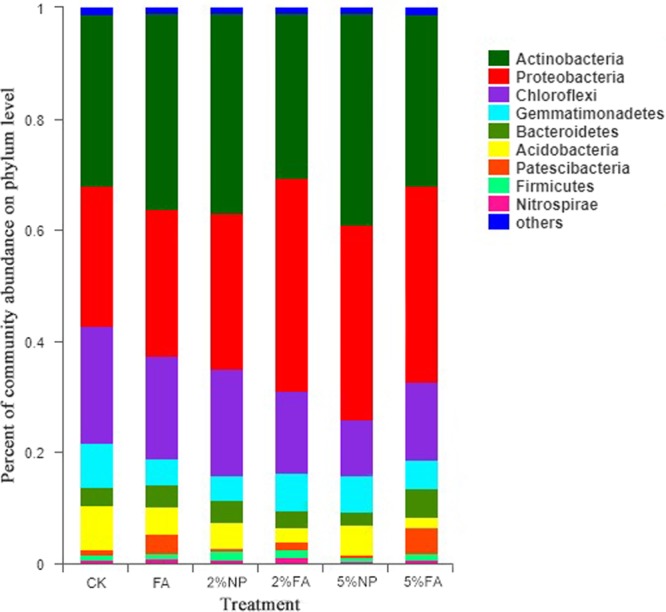
Relative abundance of the dominant microbial at phylum level in different soil samples.

**Figure 4:**
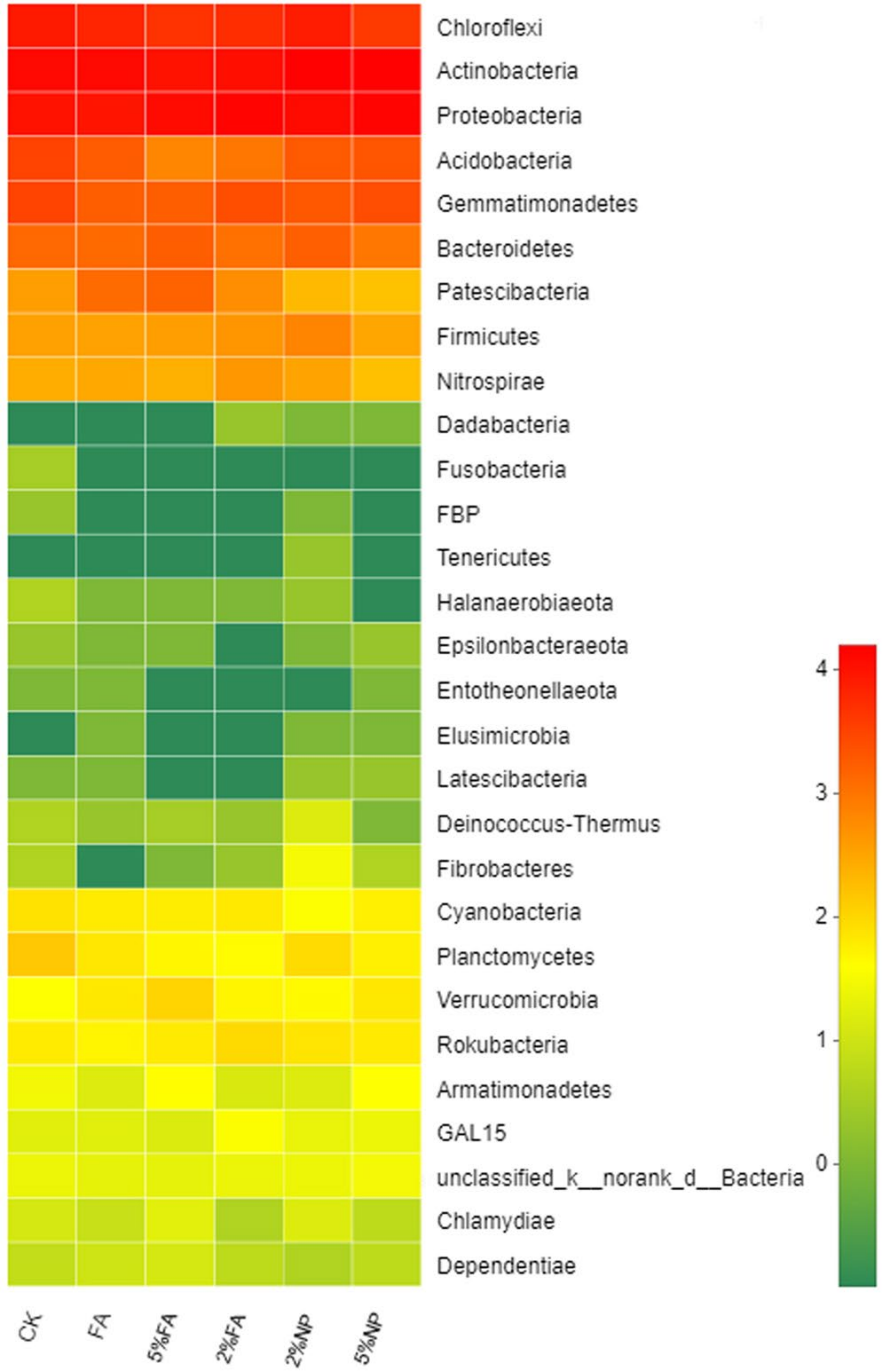
Heat map of microbial in phylum genera. The brightness of each grid represented the percentage of each microbial genus among total soil microbial.

The comparison of different remediation methods (Fig. 3) showed that although the microbial species in soil were similar at the phylum level, the abundance of microbial community changed considerably. The main phylum community in these samples were *Actinobacteria* (29.70–38.20%), *Proteobacteria* (25.14–38.35%), *Chloroflexi* (10.08–21.08%), *Gemmatimonadetes* (4.60–7.85%), *Bacteroidetes* (2.36–5.16%), *Acidobacteria* (2.36–5.16%) in decreasing order. The abundance of *Chloroflexi* community in soil decreased, whereas the abundance of *Actinobacteria* and *Proteobacteria* increased substantially. Compared with FA, the addition of materials promoted the growth and distribution of *Proteobacteria* community but inhibited the existence of the *Chloroflexi* community. No substantial difference was observed between soil microbial communities treated with FA, 2% FA and 5% FA. The heat map of microbial community distribution (Fig. 4) showed that adding HMS can increase the biomass of soil bacteria. Co-remediation considerably increased the types and abundance of microbial communities, especially soil treated with 2% FA. However, the effect of 5% addition on soil microbial community was weaker than that of 2%.

The microbial functions in all soil samples were classified (Fig. S2 in Supplementary Material), and the first seven functions with higher functional abundance were as follows: amino acid transport and metabolism; general function prediction only; energy production and conversion; transcription; carbohydrate transport and metabolism; cell wall/membrane/envelope biogenesis; inorganic ion transport; and metabolism.

### Effects of co-remediation on the distribution of Pb and the biochemical characteristics in *Festuca arundinacea*

#### The content of Pb in shoot and root of *Festuca arundinacea*

Large amounts of Pb accumulated in the shoot and root of *Festuca arundinacea*, and the concentration of Pb that accumulated in the root was higher than that in the shoot (Fig. S3a in Supplementary Material). Comparing concentrations of Pb in roots under different treatments, the co-remediation promoted the absorption of Pb in the root, and the remediation effect of 2% FA was better than that of 5% FA. The effect of co-remediation on the concentration of Pb in the shoot was not obvious. The accumulation of Pb in shoot of *Festuca arundinacea* under FA remediation was slightly higher than that under other methods. Pb content, BCF, and TF are three key parameters used to evaluate Pb accumulation efficiency in plants^35^. Figure S3b in Supplementary Material shows BCF and TF of *Festuca arundinacea* under different restoration conditions. BCF and TF of Pb in all treatments were lower than 1.0, thereby indicating that the transportation of Pb up to the ground was limited, and the root of *Festuca arundinacea* was where Pb was concentrated.

#### Enzyme activity of *Festuca arundinacea*

In this study, chlorophyll, SOD, POD, CAT and MDA were used as indicators of oxidative damage and stress resistance in plants. Figure S4 in Supplementary Material shows that the content of SOD decreased with the application of materials, whereas the content of CAT showed the opposite trend of SOD. In addition, the content of POD and MDA decreased under 2% FA treatment.

#### Distribution of Pb in subcellular of *Festuca arundinacea*

Analyzing the subcellular distribution of Pb was essential for clarifying the accumulation ability and the tolerance mechanism of *Festuca arundinacea*^36^. The results of subcellular distribution (Fig. S5 in Supplementary Material, Table 2) showed that Pb existed in the cell walls of roots and leaves in all treatments. However, no significant difference was observed in the accumulation ratio of Pb in subcells under different material treatments, and the proportion of organelles and soluble components were approximately the same. The concentration distribution (Table 2) showed that Pb migrated more to the leaves under FA method, and the inhibition effect of 2% addition on the distribution of Pb in subcellular cells was weaker than 5%. Pb was abundantly enriched in the cell wall of leaves, and almost no enrichment of Pb was observed in the organelles of leaves.

**Table 2:** Distribution of Pb in subcellular of *Festuca arundinacea* (mg/kg).

	root			shoot		
	Fcw	Fo	Fs	Fcw	Fo	Fs
FA	1322.80	126.62	120.93	189.30	5.69	29.50
2%FA	3218.21	145.16	147.60	93.48	2.45	20.96
5%FA	2270.22	131.55	124.60	90.66	2.12	19.02

## Discussion

### *Festuca arundinacea* is important contributor to the removal of Pb in soil

In the process of combined remediation, *Festuca arundinacea* played a role in removing Pb from soil, because it can remove Pb in soil through a series of behaviors, such as absorption and transportation^37^. As shown in the present study, Pb accumulated in roots and had a low translocation to shoot, which was consistent with previous conclusions^38^. Wang *et al*.^39^ and Chen *et al*.^40^ found that in different concentrations of polluted solution, Pb accumulated in roots of *Salix integra* and poplar, and our experimental results were consistent with this result. The first step of Pb accumulation in plants is to be absorbed by roots, which means roots play an important role in Pb resistance and accumulation. This phenomenon of root enrichment has two cause, namely, low Pb mobility of *Festuca arundinacea* and Pb bound by specific root tissue. These functions avoid the negative effects of Pb on photosynthesis, membrane ion permeability, and mineral nutrient uptake in plants.

**Figure 5:**
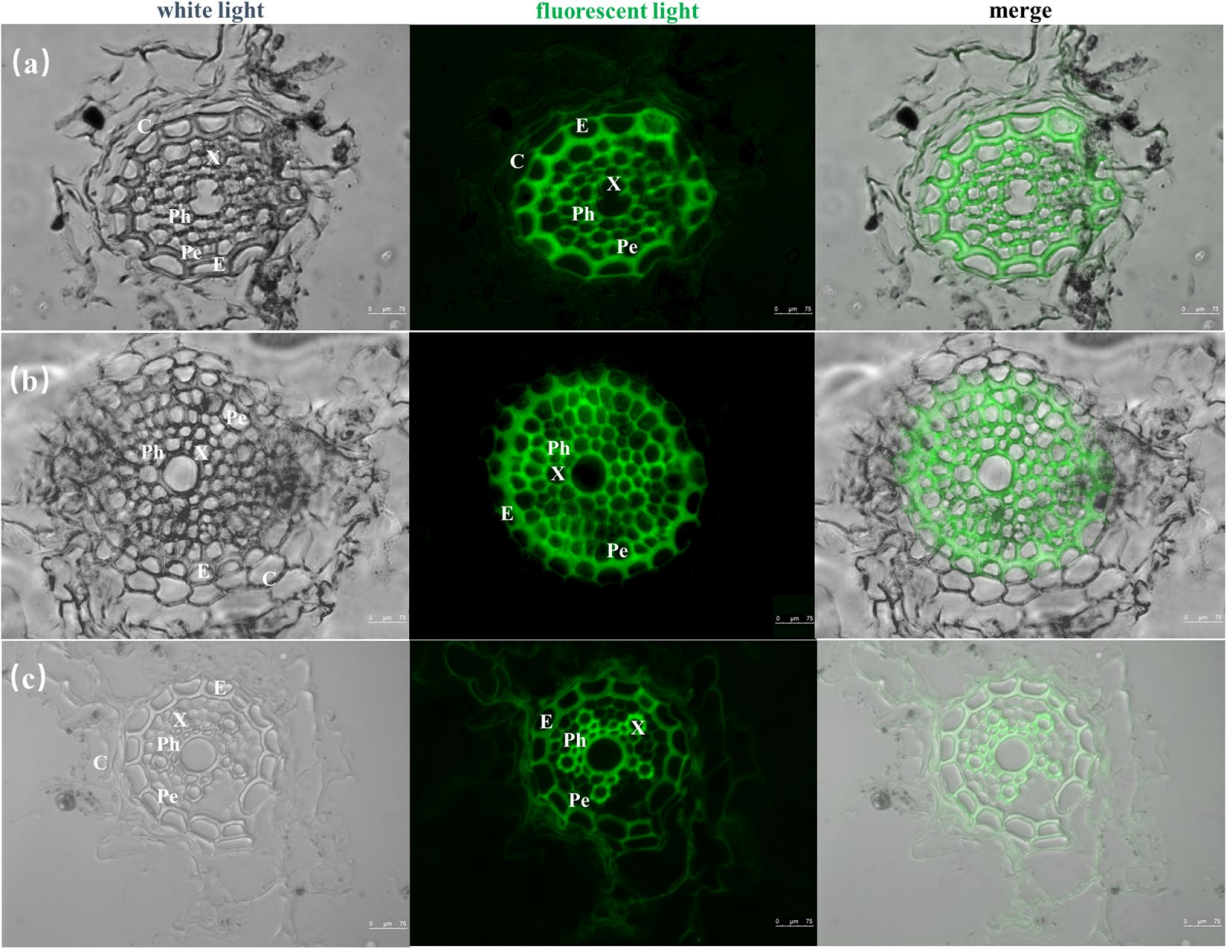
Pb distribution in the root tissues of *Festuca arundinacea*. (**a**) CK; (**b**) 2% FA; (**c**) 5% FA. C- cortex; E-endodermis; X-xylem; Ph-phloem; Pe-pericycle.

Root is an important barrier that protects plants from Pb damage. In order to determine the important mechanism that *Festuca arundinacea* can absorb Pb and protect itself from toxicity, we analyzed the fluorescence labeling of root cells of *Festuca arundinacea*. Figure 5 shows that the fluorescence intensity of the cell wall was higher than that of the cytoplasm, and that of middle column cell wall was higher than that of the cortex cell wall. The cell wall is the main storage site of the root system of *Festuca arundinacea*. The cell wall is considered the first barrier that that protects plant protoplasts from Pb. The cell wall is the first cell structure to be in contact with Pb, and storing more Pb in the cell wall protects the protoplast from damage^41^. Pb is often located in inactive regions, such as vacuoles or plasmids, to maintain the basic biological functions of roots^42^. In our study, Pb was primarily stored in the cell wall, followed by soluble components. The accumulation of Pb in cell walls can reduce the content of Pb entering organelles such as chloroplasts and mitochondria, which helped reduce toxicity of cells and maintained normal metabolic activities in cells^43^. Vacuole, as the central storage compartment in FA cells, could also store excessive Pb ion^44^. The storage function of the vacuole to Pb can reduce the activity of free lead ions and further reduce the toxic effect of Pb on organelles^36^.

Cell walls contain various polysaccharide components and proteins, which have many groups that can coordinate with Pb ions. Pb can bind with carboxyl and other functions in cell wall^23^. Pb ions unite with the ligands on the cell wall and form the precipitation^45^. After precipitation on the cell wall of *Festuca arundinacea* root system, the concentration of ions in cell protoplasm decreases, which reduces the damage of organelles caused by Pb. The result of fluorescence labeling was consistent with that of subcellular analysis. The cell walls of the root and leaf tissues of *Festuca arundinacea* had evident fluorescence phenomena, thereby confirming that the cell wall is an important site for the storage of Pb in *Festuca arundinacea*^46^. The precipitation of Pb on the cell wall of *Festuca arundinacea* can reduce the concentration of Pb ion in the protoplast, which had less damage to the protoplast or organelle. The epidermis and cortex, the outer layer of the root, can prevent the diffusion of Pb from the ectoplasma to the vascular system. Pb enters the root protoplasm of *Festuca arundinacea* before it enters the xylem, from which it can be transported to the aboveground tissues^47^.

**Figure 6:**
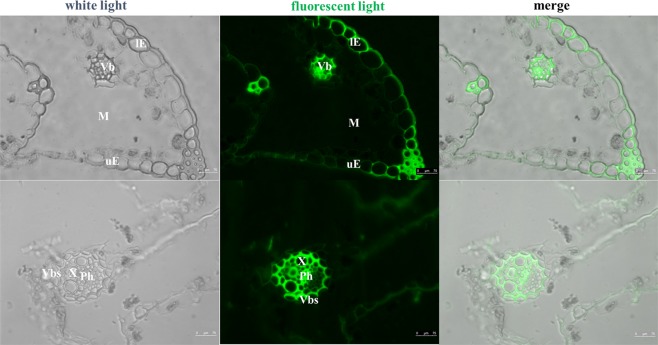
Pb distribution in the leaf tissues of *Festuca arundinacea*. (**a**) CK; (**b**) 2% FA; (**c**) 5% FA. lE- lower epidermis; uE-upper epidermis; M- mesophyll; Vb- vascular bundle; Vbs- vascular bundle sheath; Ph- phloem; X- xylem.

Although *Festuca arundinacea* can accumulate a large amount of Pb in the root, we found that there is also a amount of Pb enrichment in the shoot of the plant. Through the analysis of the enzyme activity of *Festuca arundinacea*, it is found that the toxic effect of *Festuca arundinacea* leaves is weak, which shows that it has a self-protection mechanism against Pb stress. The concentration of Pb in leaves treated with 2% FA was high. Thus, we studied the fluorescence distribution of Pb in *Festuca arundinacea* leaves treated with 2% FA (Fig. 6). The epidermis (upper and lower epidermis), mesophyll and vascular bundles of leaves showed fluorescence phenomena. The vascular bundles in leaves of *Festuca arundinacea* had high fluorescence intensity and a large fluorescence area. The fluorescence in upper epidermis was strong, whereas that in lower epidermis cells was weak. This finding suggested a possible mechanism of Pb excretion from leaves of *Festuca arundinacea*, which can alleviate toxicity of Pb to cells^48^. Based on the parts of roots and leaves of *Festuca arundinacea*, plants can enrich Pb in non-functional tissues and distribute Pb in unimportant cell components. Consequently, *Festuca arundinacea* has strong tolerance to high concentration of Pb, and its toxicity is weak after Pb adsorption. This method of stress resistance is an important mechanism that allows *Festuca arundinacea* to be used in the remediation of soil contaminated with a high concentration of Pb, which can ensure that *Festuca arundinacea* can still grow normally in polluted soil while enriching numerous Pb elements.

### Heat modified sawdust promote the stability of Pb in soil and the extraction of Pb from *Festuca arundinacea*

Water-soluble metal, exchangeable metal, and acid-soluble metal refer to metal existing in ion form or combining with carbonate, that easily accumulate in biological bodies. The F1 fraction of Pb in amended soils decreased by 24.72–31.05% after the addition of 1:2 mixed HMS to the contaminated soil, as shown in our previous study. Lu *et al*.^49^ attributed the changes to the stabilization of metals by biomass materials. However, an increase in water-soluble, exchangeable and acid-soluble components of Pb, which may be due to the interaction between remediation materials and root exudates of *Festuca arundinacea*, was observed in the present study. HMS may interact with root exudates to form carbonate in phytoremediation, and the combination of carbonate and Pb increases the proportion of PbCO_3_ in polluted soil. Figures 2 and S1 show that the value of F1 in 2% FA was higher than that in 2% NP, whereas that in 5% FA was lower than that in 5% NP. The passivation of Pb by remediation materials and the activation of Pb by plants existed simultaneously. Moreover, the passivation effect of 5% material addition on Pb was higher than that of 2%, which can also be seen from Fig. S1. The figure showed that the value of F1 in 2% FA was higher than in 2% NP and that in 5% FA was lower than in 5% NP. In addition, the passivation effect of 5% additive on Pb was stronger than the activation effect of plants on Pb in the system with plants. Based on the different trends of fraction, we conclude that the dominant mechanism of co-remediation was different. The decrease of bioavailable-Pb indicated that the stabilization of material and *Festuca arundinacea* was the dominant mechanism of treatments under 5% FA, whereas the increase of bioavailable-Pb indicated that the uptake of Pb by *Festuca arundinacea* was the dominant mechanism of 2% FA co-remediation.

When materials and plants coexist, the role of biomass affects the absorption process of heavy metals and nutrients by plants^50^. The stabilization of HMS to Pb in soil may involve many complex mechanisms, and the addition of materials may change the concentration of Pb in plants, especially in different parts of *Festuca arundinacea*^51^. The concentration of Pb in root of *Festuca arundinacea* under co-remediation was higher than others, which indicated that co-remediation promoted the accumulation of Pb in roots and enhanced the phytoremediation ability of *Festuca arundinacea*. The strengthening effect of HMS on root restrained the upward migration of Pb and played a positive role in protecting shoot tissue. Anning *et al*.^52^ showed that soil amelioration could reduce the binding of metal cations to extracellular cation exchange sites in the cytoplasm and cell wall, then increase the root flux of metal through the cytoplasm into the column. Pb, a non-essential element, has no specific transport channel or transporter in plants, which leads to the need for lead to enter plants through the channel of transport of essential elements^53^. HMS can change the concentration of essential elements in soil. The presence of Fe, Ca, and Mg in materials may stimulate the transport and uptake of essential elements by plants, and this process promotes the uptake of Pb by plants and its entry into root cells. The fluorescence intensity of the middle column of root system (Fig. 5b) was highest under 2% FA treatment, which means that 2% FA co-remediation could promote the uptake of Pb by *Festuca arundinacea*.

HMS can stimulate the root of *Festuca arundinacea* and increase the absorption of Pb by the root of *Festuca arundinacea*. At the same time, the presence of materials can also reduce the Pb toxicity of *Festuca arundinacea*. The persistent functional groups and free radicals on the surface of the remediation materials may help plants scavenge reactive oxygen species, and the regulation of materials may lead to the reduction of metal oxidative damage to plants^54^. HMS addition reduced the accumulation of hydrogen peroxide, inhibited the damage caused by excessive ROS production to plants, and alleviated the damage caused by Pb to *Festuca arundinacea*^53^. In addition, the content of POD and MDA decreased under 2% FA treatment, which indicated that the process of reducing metal-induced toxicity was concentration dependent, and the appropriate content of remediation materials could reduce the oxidative damage of heavy metals effectively^53^. The results of fluorescence labeling also showed that the remediation materials had considerable protective effects on *Festuca arundinacea*. Numerous granular crystals and cell damage appeared in the cortex of root under FA treatment (Fig. 5a) due to the toxicity of Pb to *Festuca arundinacea* cells^55^. The root cells of *Festuca arundinacea* were intact, and the different cell layers could be clearly observed in the 2% FA treatment (Fig. 5b). When the addition of material increased to 5%, the lines in the middle column of *Festuca arundinacea* were irregular and blurring (Fig. 5c). Salt stress induced by HMS at high concentration causes osmotic pressure imbalance, which damages cell structure^56^. Although the damage degree of the root system differed, the integrity of central column and internal tissue was much higher than that of epidermis and cortex, which indicates that *Festuca arundinacea* limited the toxicity to relatively unimportant tissue structure to reduce Pb damage.

A comprehensive analysis of the results of Pb concentration and enzyme activity in *Festuca arundinacea* showed that 2% of the materials promoted the accumulation of Pb in the shoot and root of *Festuca arundinacea*, and had a strong protective and promoting effect on the growth and development of *Festuca arundinacea*. The mixture of SA and SB in remediation passivated the Pb in soil and protected and promoted the growth of *Festuca arundinacea*. It also improved the ability of *Festuca arundinacea* to tolerate and absorb Pb. Thus, the mixture material had a good remediation effect on high Pb pollution in soil.

### Co-remediation improve soil environment

The goal of co-remediation of soil Pb pollution includes not only the reduction of Pb content, but also the improvement of soil environment. Soil organic matter content, microbial community diversity and soil enzyme activity are important indexes to evaluate soil environment^23,57,58^.

Addition of HMS can increase the content of DOM in soil, but characteristics and functions differ between biomass source DOM and natural soil DOM^59^. Phytoremediation can change the content and composition of DOM in soil. The DOM content of soil treated with 2% FA and 5% FA was higher, whereas the A_250_ of 2% FA and 5% FA was lower, and the amount of macromolecule organic matter in the soil planted with *Festuca arundinacea* was less than that in soil without plants. Co-remediation could transform macromolecular organic matter into small molecular compounds, and the dissociation of macromolecule organic matter occurred during phytoremediation. Compared with macromolecule DOM, small molecule DOM has more binding sites, and the complexation ability of Pb with DOM decreases considerably with increasing molecular weight of DOM^60^. DOM is the carrier of Pb, but the adsorption of Pb by DOM coexists with that by soil colloids. The content of non-soluble organic matter in soil is absolutely superior to that of soluble organic matter. If Pb ions are adsorbed by non-soluble organic matter in soil particles, then the occurrence of co-migration will be greatly reduced^61^. According to the value of A260, the combined remediation process reduced the content of hydrophobic components in DOM, which also promoted the adsorption and fixation of Pb in soil. Kaiser^62^ found that soil adsorption of hydrophobic DOC decreased with increasing soil organic matter content. Therefore, the increase of DOM and the change of soil organic matter composition during co-remediation may explain the increase of Pb absorption and stabilization.

On one hand, co-remediation can reduce the content of Pb in soil and increase the activity of enzymes and organic matter. On the other hand, it is beneficial to the promotion of the growth of tolerant microorganisms and improves the release of more substances by microorganisms to enhance the ability of plants to remediate Pb. Studying the effects of co-remediation on microbial communities in rhizosphere soil helps elucidate the effects of environmental changes on microbial communities, and to determine the role of microbial communities in co-remediation. No substantial difference was observed between soil microbial communities treated with FA, 2% FA and 5% FA, which indicated that the effects of plant existence on microbial communities were complex and had many interactions between them. The cultivation of *Festuca arundinacea* had a positive stimulating effect on *Actinobacteria* and *Proteobacteria* communities. Genome studies have shown that *Actinobacteria* and *Proteobacteria* communities are involved in carbohydrate transport and metabolism, and inorganic ion transport and metabolism; *Bacteroidete* communities are rich in glycosyltransferases and degrading enzymes. The combination of these functional bacteria can stimulate plant growth and improve the ability of *Festuca arundinacea* to extract Pb^63^. The presence of plants can improve the diversity of soil microbial community, and the addition of HMS can also contribute to microbial abundance and activity by providing space and environment for microorganisms^64^. Shamim Gul *et al*.^57^ found that the addition of biochar contributed to the enrichment and activity of microorganisms, the pores on the material surface provided habitat for microorganisms. However, the effect of 5% addition on soil microbial community was weaker than that of 2%, which indicated that excessive HMS had some potential side effects on soil microbial community. The response of soil bacterial community (diversity and structure) to the amount of remediation materials was proportion dependent. Manyun Zhang *et al*.^58^ also found that 6.0% w/w of biochar could reduce the diversity of soil bacterial community. In addition to passivizing Pb, the application of remediation materials can also absorb soil nutrients^53^. Therefore, higher addition may reduce the bioavailability of nutrients in soil, which restricts the reproduction of soil bacteria and affects the soil remediation performance^65^.

**Figure 7:**
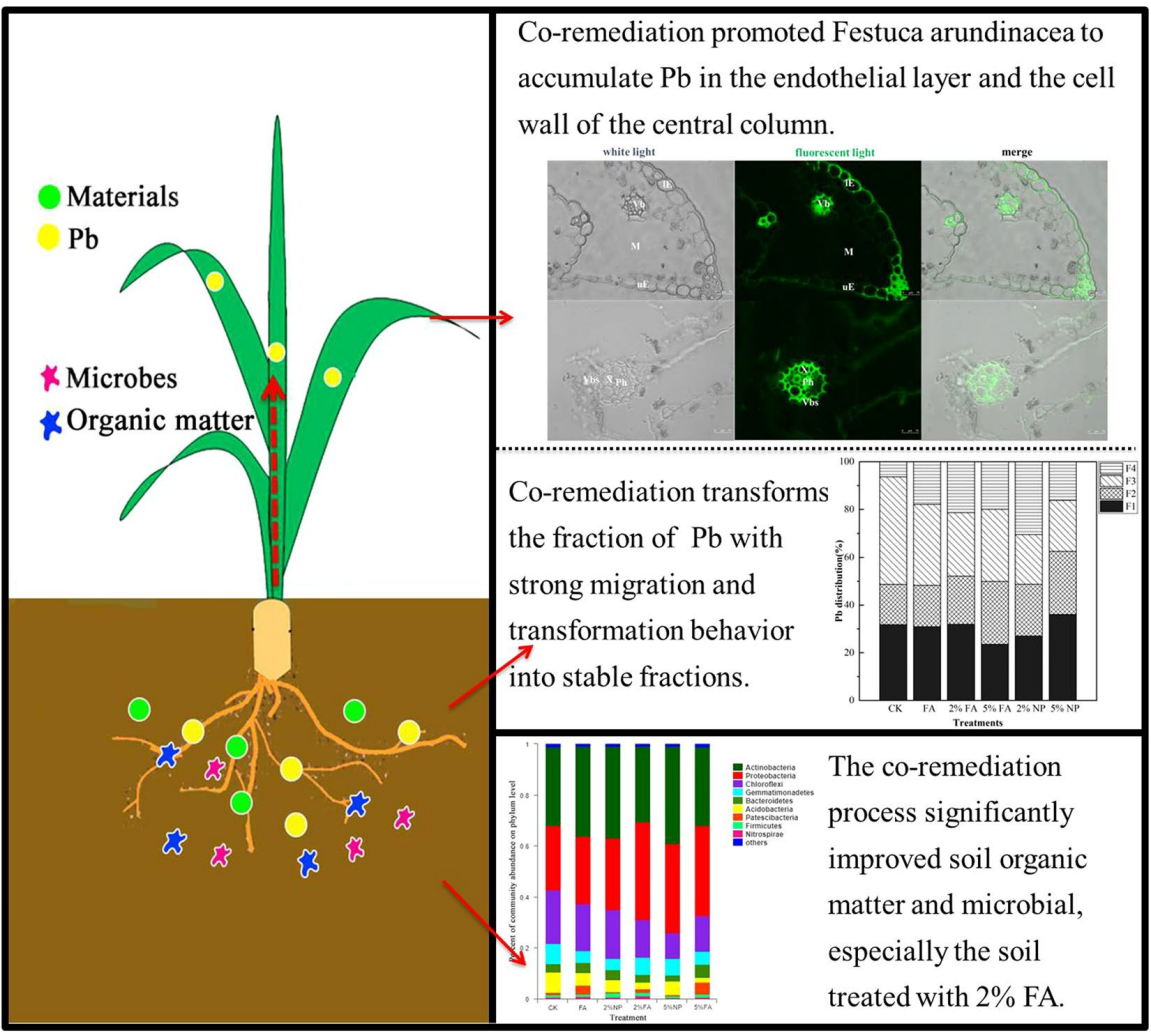
Effect and process of Pb contaminated soil remediation by Festuca arundinacea and heat modified sawdust.

Compared with single remediation, co-remediation, especially 2% FA remediation method, had evident advantages. Co-remediation promoted the adsorption of Pb by *Festuca arundinacea* and improved the soil environment. The existence of material promoted the fixation of Pb by plant roots, especially cell walls, and inhibited the upward migration of Pb. The improvement of soil environment can also promote better plant growth and improve the ability of Pb phytoremediation (Fig. 7).

## Conclusion

Co-remediation of *Festuca arundinacea* and the mixture of sawdust ash and sawdust biochar reduced the total amount of Pb in soil. Co-remediation of 5% additive material and *Festuca arundinacea* had the best effect on the removal of Pb. The leaching toxicity and bioavailability of Pb in soil were reduced with the addition of remediation materials. Co-remediation reduced the percentage of Pb in acid extractable fraction, and transformed more of Pb into organic matter-bound and residual forms. Co-remediation changed the types of soil organic matter. The dissociation of macromolecular organic matter into micromolecular organic matter was more conducive to the complexation and stability of Pb. The effect of 2% FA co-remediation on soil microbial community type and abundance was greater than that of 5% FA, which indicated that excessive material may reduce the bioavailability of nutrients in soil. Although the accumulation of Pb in the shoot of *Festuca arundinacea* treated with 2% FA was higher than others, the damage degree of *Festuca arundinacea* treated with 2% FA was lower. The combination of 2% material addition and *Festuca arundinacea* promoted the adsorption of Pb by plants and protected the growth of plants. Co-remediation can promote *Festuca arundinacea*’s accumulation of Pb in the endothelial layer and the cell wall of the central column, and the accumulation of Pb in the cell wall of root was improved by the 2% FA treatment. Heat treated biomass material can strengthen plant roots, inhibit the trend of Pb migration to shoots, and play a positive role in protecting plant shoots.

## Supplementary information

Supplementary information.

## Data Availability

All data used in this publication will be provided upon request by the corresponding author.
