# Supplementary material for: Co-remediation of Pb Contaminated Soils by Heat Modified Sawdust and Festuca arundinacea
Source: Sci Rep. 2020 Mar 13;10:4663. doi: 10.1038/s41598-020-61668-x (PMC7069995; doi:10.1038/s41598-020-61668-x)
Supplement: Supplementary file 1 — Supplementary information. [file 41598_2020_61668_MOESM1_ESM.docx]

**Supplementary Material**

**Co-remediation of Pb Contaminated Soils**

**by Heat Modified Sawdust and *Festuca arundinacea***

Yan Zhang^1^, Xuemei Wang^1^, Hongbing Ji^1, 2*^

1. Beijing Key Laboratory of Resource-oriented Treatment of Industrial Pollution, School of Energy and Environmental Engineering, University of Science and Technology Beijing, Beijing, 100083, China

2. Beijing Municipal Key Laboratory of Resource Environment and GIS, College of Resource Environment and Tourism, Capital Normal University, Beijing 100048, China

**Correspondence author: Prof. & Dr. Hongbing Ji, University of Science and Technology Beijing, Xueyuan Road No.30, Haidian District, Beijing-100083, P.R. China. E-mail address: e-mail: ji.hongbing@hotmail.com, Tel: +86-10-62332750; Fax: +86-10-62332750*

# Materials and methods

### Sample determination and analysis

### Pb content and fractions in soil

After removing rocks and impurity, soil samples were air-dried and ground to pass through a 200-mesh sieve. The mixed acid (HNO_3_/HCl = 3:1) treated soil sample (0.1 g) was digested using a microwave digestion system (Michem DR-24 Infrared Digester, China). The total concentration of Pb was determined by ICP-OES (Varian 720/-ES).

The detailed experimental steps of SPLP are as follows: the soil samples were leached by the solutions treated with H_2_SO_4_ and HNO_3_ (2/1 w/w) at pH 3.20 to simulate acid rain leaching process (10 L/kg, liquid/solid) and agitated in a rotary oscillator at 30 ± 2 r/min for 18 h (China, HJ/T299-2007). After the leaching procedure, the samples were centrifuged and filtered for analysis. The DTPA extraction method is used to extract the bioavailable state of soil heavy metals, and the steps are described as follows: shaking 5 g of soil samples with 10 mL solutions containing 0.005 M DTPA, 0.01 M CaCl_2_, and 0.1 M TEA (Mtriethanolamine) at pH 7.3 in a shaker (THZ-C-1) at 180 rpm for 2 h. The mixture was centrifuged and filtered for analysis. In BCR method, water-soluble and exchangeable metals (F1, 0.11 M CH_3_COOH), reducible metals bound to Fe and Mn (F2, 0.1 M NH_2_OH·HCl, pH 2), oxidizable metals bound to organic matter and sulphides (F3,8.8 M H_2_O_2_ and 1 M NH_4_OAc, pH 2), and residual metals bound to silicate minerals (F4,total content-F1-F2-F3) were extracted. All the extractions were centrifuged and filtered for analysis.

Among the four fractions extracted in BCR sequential extraction, water-soluble and exchangeable metals (F1) are small but have strong mobility and bioavailability ^1^. Reducible metals bound to Fe and Mn (F2) and oxidizable metals bound to organic matter and sulphides (F3) are readily released when environmental conditions change to produce bioavailability, so they are classified as potentially toxic ^2^. While, residual metals bound to silicate minerals (F4) is stable and this form has little contribution to the migration and bioavailability of PTEs in soil ^3^.

### Pb content in *Festuca arundinacea*

The plant was treated according to the method described by Zhang^2^. Plant samples were divided into shoot and root parts, and the shoot part was divided into stems and leaves^2^. The plants were heated for 0.5 h under 105 °C in paper bags, dried until constant weight in an oven at 70 °C, and then passed through a 100-mesh sieve for further experiment^2^. The mixture (HNO_3_/H_2_O_2_ = 5:1) treated plant (0.2 g) was digested using a microwave digestion system (Michem DR-24 Infrared Digester, China). The concentrations of Pb were determined by ICP-OES (Varian 720/-ES)^2^.

The analysis of Pb content in the subcellular of *Festuca arundinacea* are as follows: Fresh root or shoot tissues (0.5g) were homogenized in 10mL pre-cooled extraction buffer (0.25mol/L sucrose, 50 mmol/L Tris-HCl (pH 7.5), 1.0mmol/L dithioerythritol). The homogenate was moved into a 50 mL centrifugal tube and were centrifuged to three cell components by differential centrifugation：cell walls fraction (Fcw) (3500g for 20 min，the precipitate), soluble fraction (Fs) (18,000g for 40 min, the supernatant solution) and organelles fraction (Fo) (18,000g for 40 min, the precipitation). All procedures were carried out at 4 ℃. The supernatant and precipitation were oven dried at 70℃ to dryness, then digested with HNO_3_-HClO_4_ (5:3, v/v). The Pb concentrations in different cell components were determined by ICP-OES.

### Assays of plant physiological indexes

Chlorophyll was extracted by the method described by Chen ^4^ that 0.1 g fresh leaf sample was shredded and placed in a mixed solution of 10 ml (acetone: ethanol: distilled water = 4.5:4.5:1) for 2 days in the darkness of 4 C. Chlorophyll contents in supernatants were analyzed spectrophotometrically (METASH UV-5500, China) following the method of Aaron ^5^ at wavelengths 663 nm and 645 nm. Peroxidase (POD) activity was determined following the modified guaiacol method ^6^. Exactly 1mL of enzyme extract was mixed with 3mL of phosphate buffer（pH 6）containing 28 μL of 0.2% guaiacol and 19 μL of 0.3% H_2_O_2_. The reaction mixture was determined immediately using an UV spectrophotometer (METASH UV-5500, China) at 470nm and change in the absorbance for 3 min was recorded for the calculation of POD activity. POD activity was expressed as units of U·g^-1^ min^-17^. Catalase (CAT) activity in plant was measured with the method described by Aebi ^8^ with slight modification. The 3 ml of reaction media contained 30 mM H_2_O_2_ in a 50 mM phosphate buffer (pH 7.0) and 0.1 ml of enzyme. The activity was estimated according to the decrease in absorbance of mixture at 240 nm with H_2_O_2_ in reaction mixture for 2 min. One unit (U) of CAT activity is the amount of enzyme dissociating 1 nmol H_2_O_2_ min^-1 9^. The superoxide dismutase (SOD) activity was assayed through the photoreduction of nitroblue tetrazolium (NBT) method ^10^. The 3 mL of solution contained 0.05mL of extract, 0.3mL of NBT, 0.3mL of EDTA-Na_2_, 0.3 mL of methionine and 0.3mL of riboflavin and 1.5mL of sodium phosphate buffer in 0.25mL of distilled water. The mixture was placed under 4000 Lx (80μmol·m^-2^·s^-1^) for 20~30min then turned off and stopped the responding, and the reaction was measured as the increase in absorbance at 560 nm^11^. No light response and no supernatant reaction were used as controls. The total activity of SOD was expressed in the enzyme unit g^-1^ FW (fresh weight). The content of malondialdehyde (MDA) was assayed by the thiobarbituric acid (TBA) method to measure membrane lipid peroxidation ^12^. The MDA in plant samples was extracted by 10% trichloroacetic acid (10 ml) and centrifugated. The 2mL of supernatant was added in 2 ml of 0.67%TBA and reacted 15min in the boiling water bath. The absorbance of the reaction was measured at 532 and 600 nm. The concentration of MDA was expressed in μmol g^−1^ FW.

Table S1 Potted Experiment Soil Physicochemical Properties

|  |  | pH | Pb  (mg/kg) | TOC |
| --- | --- | --- | --- | --- |
| Soil sample |  | 6.56 | 30870 | 5.74% |
| national environmental quality standard | Grade I | natural background | 35 | - |
|  | Grade II | <6.5 | 250 | - |
|  |  | 6.5~7.5 | 300 | - |
|  |  | >7.5 | 350 | - |
|  | Grade III | >6.5 | 500 | - |

* Grade I is natural ecological background value of protected area；Grade II is soil limit value to ensure the normal agricultural production and to maintain the plant health; Grade III is soil critical value to maintain the normal growth of plants.

Table S2 Physiochemical characteristics of sawdust ash (SA) and sawdust biochar (SB)

| Material | textural properties | | |  | component analysis (%) | | | | pH |
| --- | --- | --- | --- | --- | --- | --- | --- | --- | --- |
|  | surface area（m^2^/g） | pore volume（cc/g） | average pore diameter(nm) |  | C | N | H | S |  |
| Sawdust ash (SA) | 8.11 | 9.74×e^-3^ | 4.80 |  | 26.63 | 1.04 | 0.46 | 0.14 | 10.21±0.02 |
| Sawdust biochar (SB) | 59.23 | 4.24×e^-2^ | 2.87 |  | 58.52 | 0.47 | 2.23 | 0.32 | 10.23±0.01 |

Table S3 Treatments of pot experiment

| Combined Treatments | Abbreviation |
| --- | --- |
| 1.5kg soil (control) | CK |
| 1.5kg soil+ *Festuca arundinacea* | FA |
| 1.5kg soil +2% w/w remediation material | 2% NP |
| 1.5kg soil +2% w/w remediation material + *Festuca arundinacea* | 2% FA |
| 1.5kg soil +5% w/w remediation material | 5% NP |
| 1.5kg soil +5% w/w remediation material + *Festuca arundinacea* | 5% FA |

Table S4 Experimental methods for speciation extraction and toxic leaching of Pb in soil

| Method | Extractant | Substances extracted | Substances abbreviation | Rif |
| --- | --- | --- | --- | --- |
| Synthetic precipitation leaching procedure (SPLP) | H_2_SO_4_ and HNO_3_ (2/1 w/w) at pH 3.20 | - | - | ^13^ |
|  | | | | |
| DPTA (Diethylenetriaminepentaacetic acid) extraction method | 0.005 M DTPA, 0.01 M CaCl_2_, and 0.1 M TEA (Mtriethanolamine) at pH 7.3 | Bioavailability of heavy metals | - | ^14^ |
|  | | | | |
| BCR (European Community Bureau of Reference) method | 0.11 M CH_3_COOH | Water-soluble and exchangeable metals | F1 | ^15,16^ |
|  | 0.1 M NH_2_OH•HCl at pH 2 | Reducible metals bound to Fe and Mn | F2 |  |
|  | 8.8 M H_2_O_2_ and 1 M NH_4_OAc at pH 2 | Oxidizable metals bound to organic matter and sulphides | F3 |  |
|  | Total content-F1-F2-F3 | Residual metals bound to silicate minerals | F4 |  |

Table S5 Description of UV-Vis Parameters

| Absorption spectral parameters | Formula | Formula parameters | Description |
| --- | --- | --- | --- |
| absorption coefficient a(λ) | a(λ)=2.303A(λ)/r | a(λ) is absorption coefficient of λ wavelength (m^-1^)，A(λ) is absorbance of λ，r is optical length (m) | - |
| SUVA_254_ | SUVA_254_=a(254)/DOC | a(254) is absorption coefficient of 254nm | characterize the aromaticity of DOM |
| SUVA_260_ | SUVA_260_=a(260)/DOC | a(260) is absorption coefficient of 260nm | characterize the content of hydrophobic components in DOM |
| A_280_ | - | A_280_ is absorbance of 280nm | characterize the aromatization degree of organic matter |
| A_250_／A_365_ | - | A_250_ and A_365_ are absorbances of 250nm and 365nm, respectively | inversely proportional to the molecular weight of DOM |

Table S6 Total concentration and removal rate of Pb in soil after the incubation experiment (after 60 days)

|  | Concentration(mg/kg) | Removal rate (%) |
| --- | --- | --- |
| CK | 30870±2948.1 | - |
| FA | 30100±2731.3 | 2.49±0.32 |
| 2% NP | 29960±2298.8 | 2.95±0.41 |
| 2% FA | 26980±2311.3 | 12.60±1.56 |
| 5% NP | 28710±2444.2 | 7.00±1.05 |
| 5% FA | 25308±2110.4 | 18.02±2.08 |


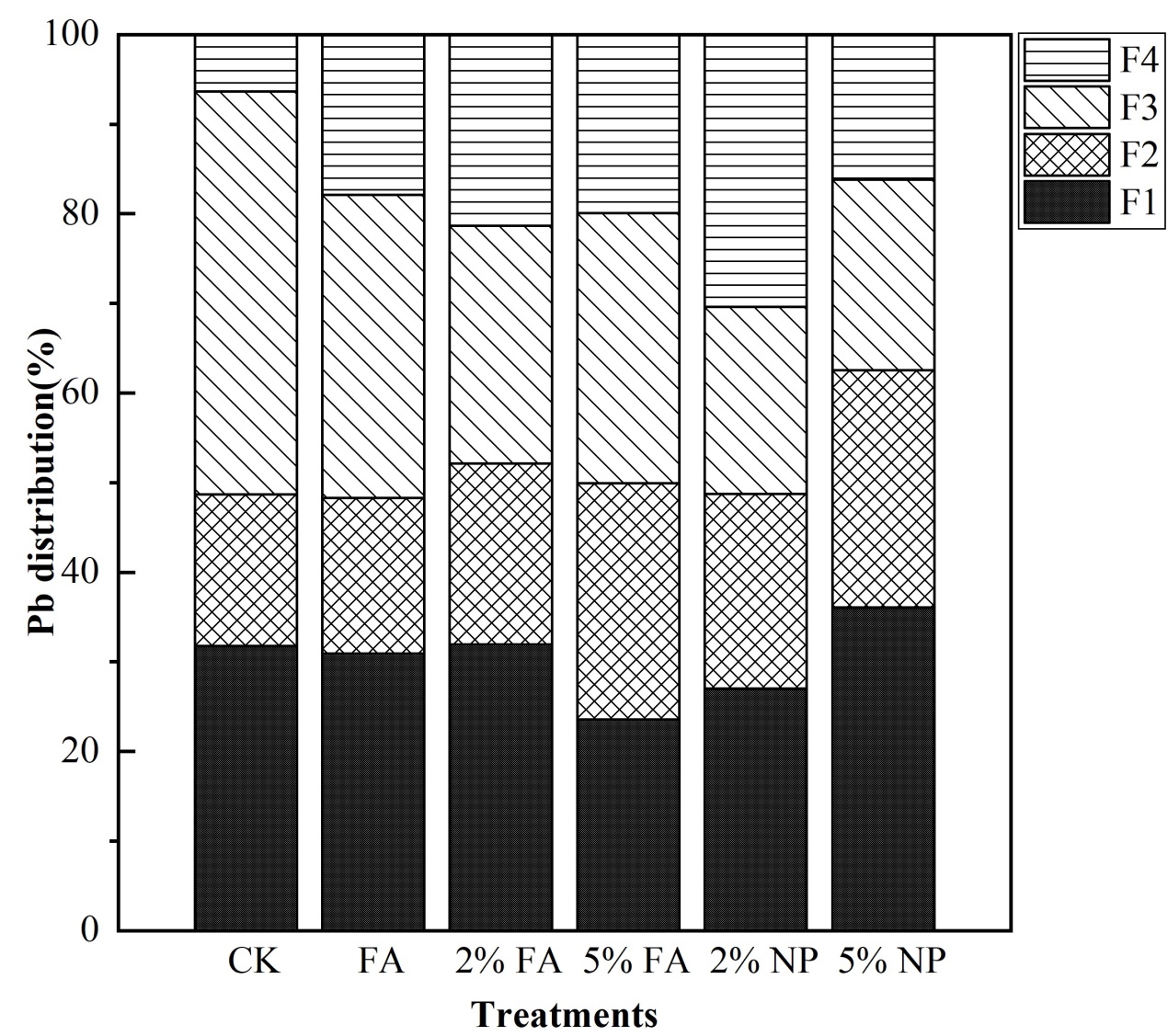


Fig. S1 Fractions of Pb in treated and untreated soils. Graphic symbols represent different types of materials to be added, while colors represent different ratios of materials to be added. F1: Water-soluble and exchangeable Pb, F2: Reducible Pb bound to Fe and Mn, F3: Oxidizable Pb bound to organic matter and sulphides, F4: Residual Pb bound to silicate minerals.


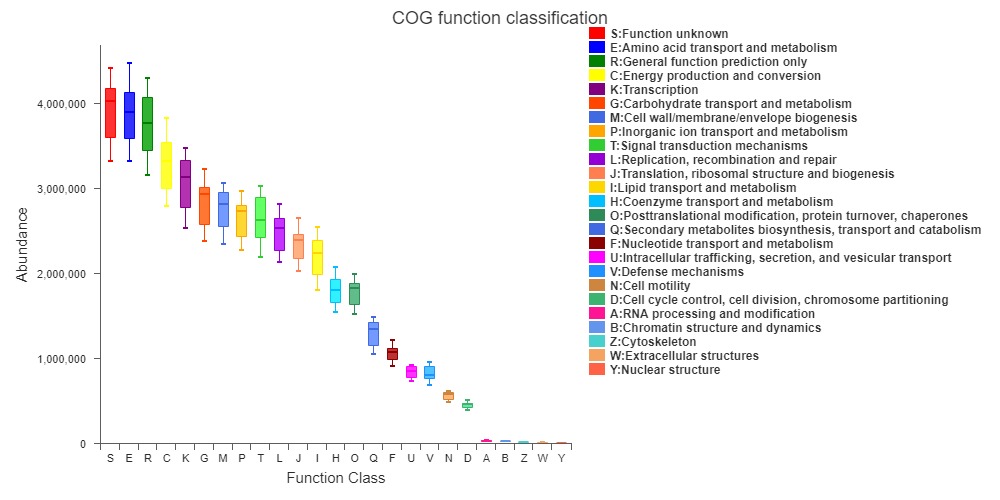


Fig. S2 Classification of Microbial Functions in All Soil Samples


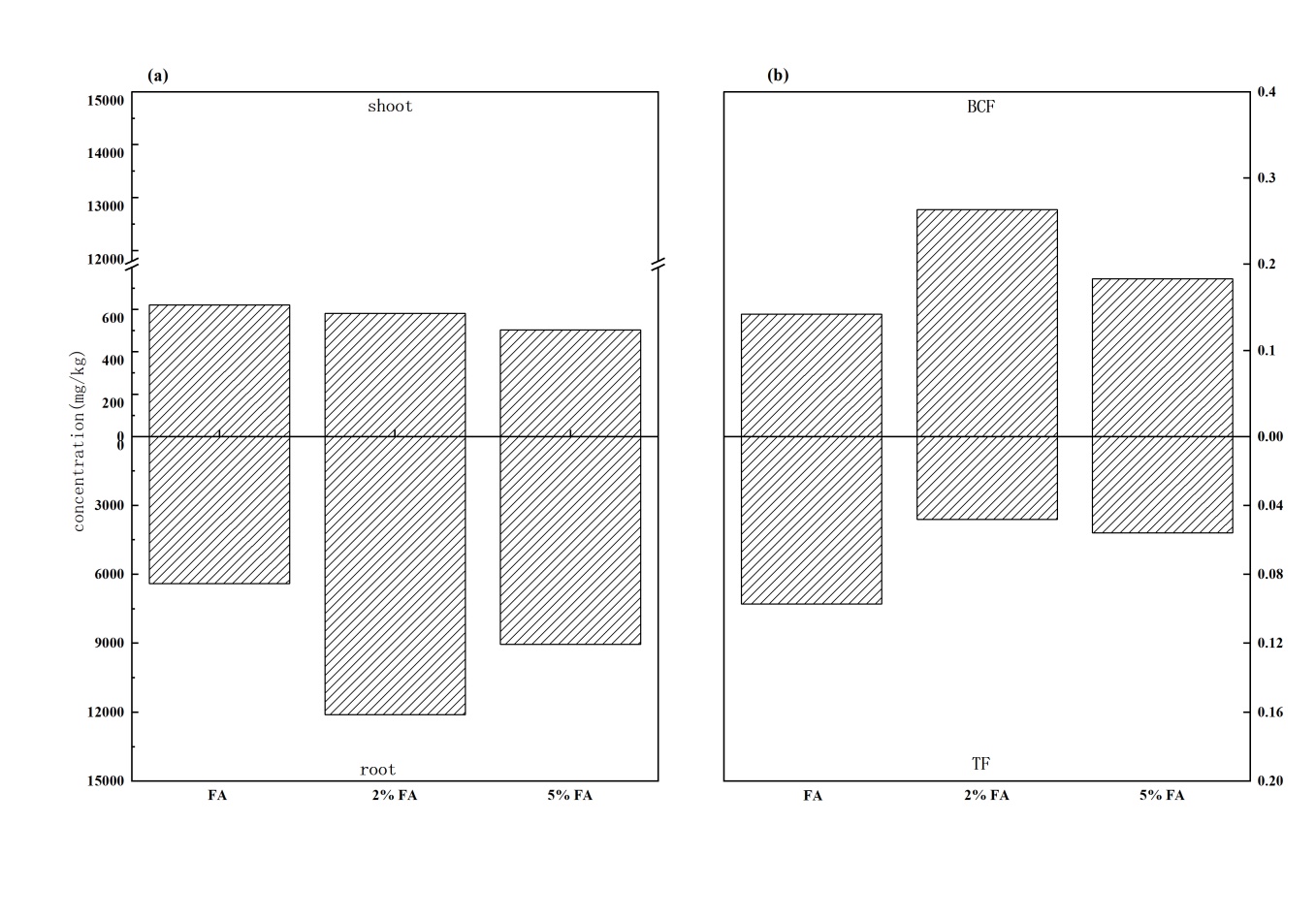


Fig. S3 The concentration, bioconcentration factor (BCF) and translocation factor (TF) for Pb of *Festuca arundinacea* under different treatments. a: Concentration (mg/kg DM) for Pb in shoot and root of *Festuca arundinacea*; b: Bioconcentration factor (BCF) and Translocation factor (TF) for Pb of *Festuca arundinacea*


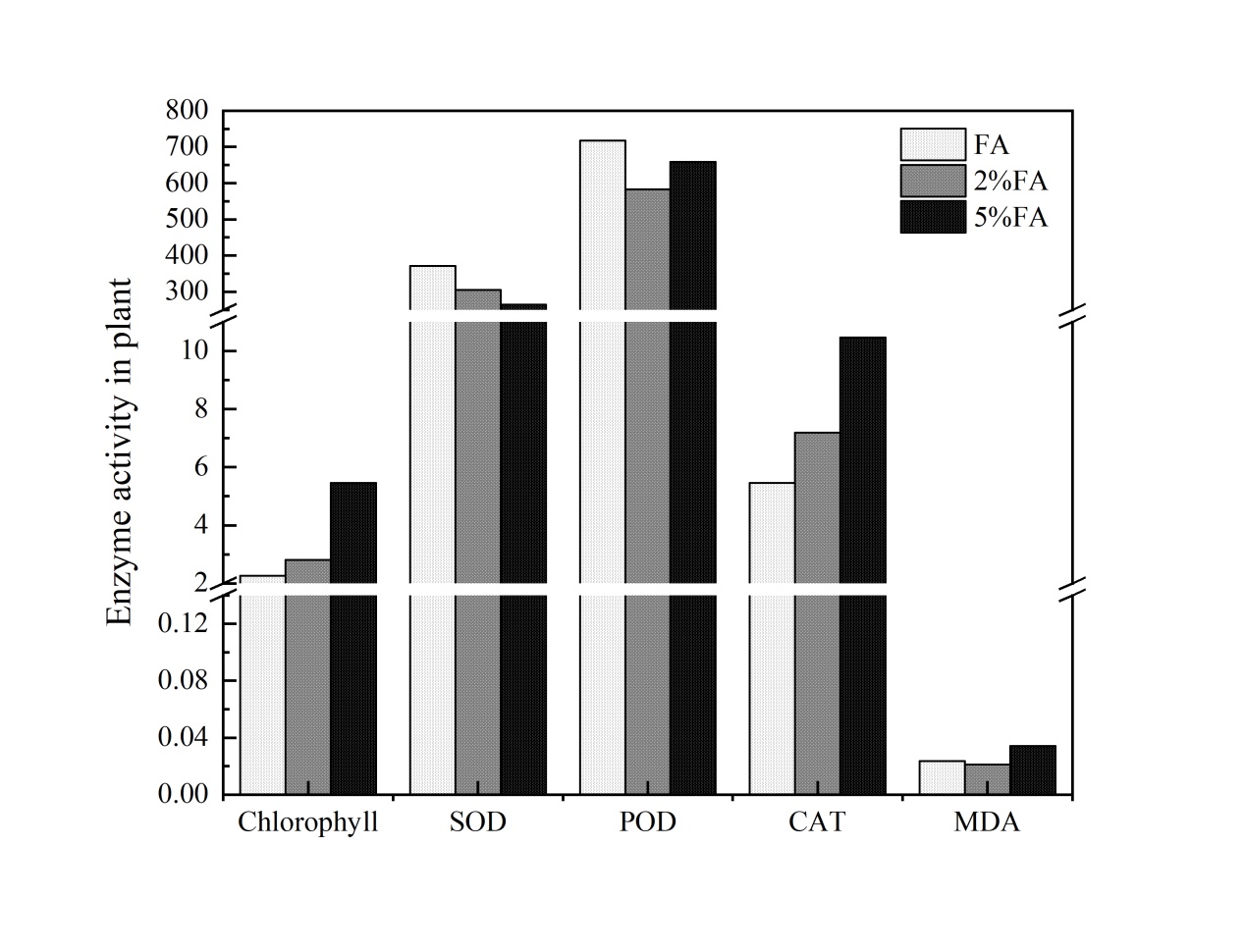


Fig. S4 Effects of different treatments on contents of Chlorophyll, SOD, POD, CAT and MDA in *Festuca arundinacea*


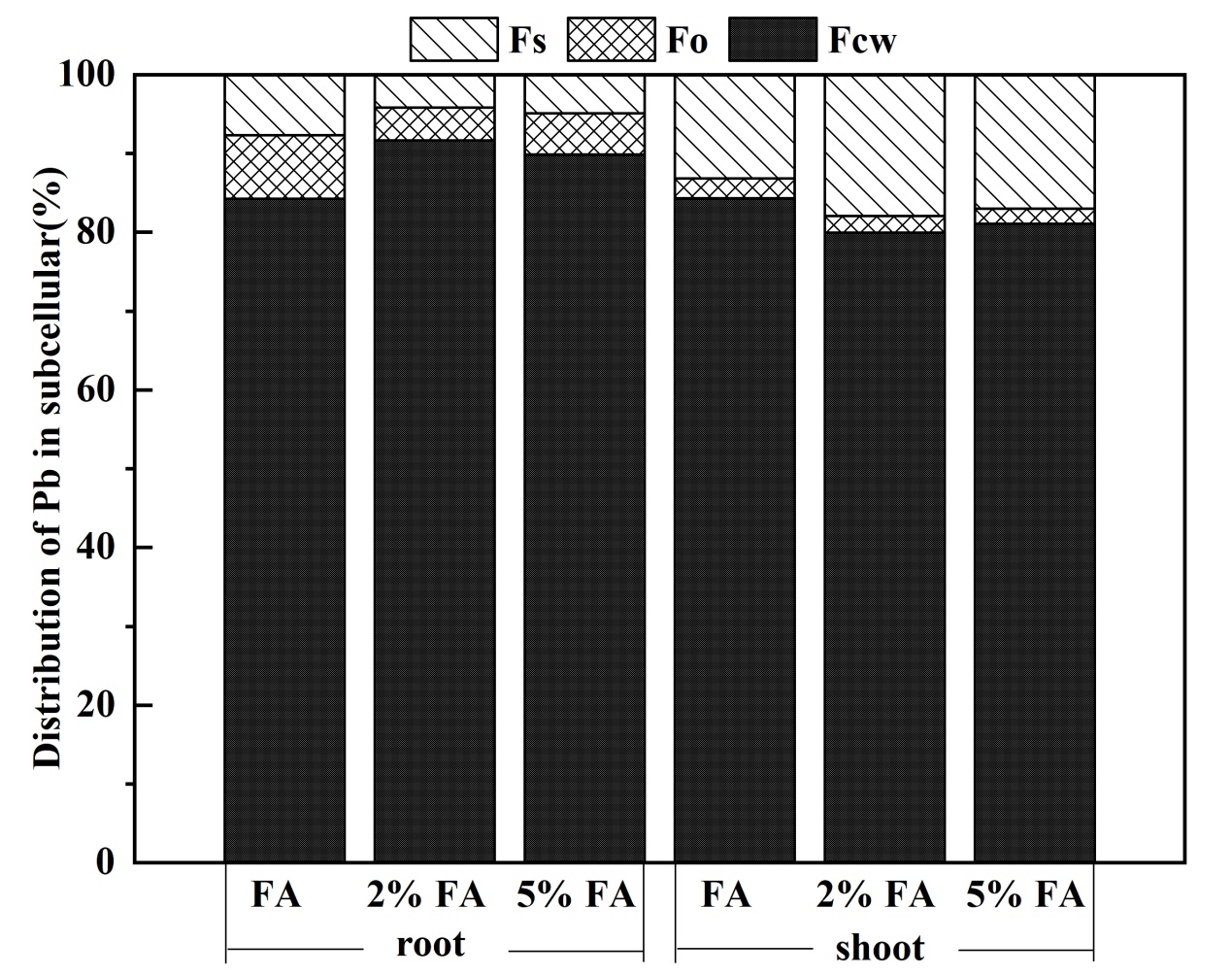


Fig. S5 Subcellular distribution of Pb in *Festuca arundinacea* root and leaf under different treatments. Fcw: cell walls fraction; Fs: soluble fraction; Fo: organelles fraction.

# Reference

1 Wen, J., Yi, Y. & Zeng, G. Effects of modified zeolite on the removal and stabilization of heavy metals in contaminated lake sediment using BCR sequential extraction. *J. Environ. Manag.* **178**, 63-69, doi:10.1016/j.jenvman.2016.04.046 (2016).

2 Zhang, Y. & Ji, H. Physiological responses and accumulation characteristics of turfgrasses exposed to potentially toxic elements. *J. Environ. Manage.* **246**, 796-807, doi:10.1016/j.jenvman.2019.06.030 (2019).

3 Wang, Z. *Study on of heavy metal pollution Characteristics and soil enzyme activities in typical mining area* master thesis, Donghua University, (2010).

4 Chen, Z., Fu, Y. & Qu, Q. Determination of chlorophyll content by the mixed method of dimethyl sulphoxide with acetone. *J. Shandong Agr. U.* **20**, 31-35 (1989).

5 Arnon, D. I. Copper enzymes in isolated chloroplasts. polyphenoloxidase in beta vulgaris. *Plant Physiol.* **24**, 1-15 (1949).

6 Fielding, J. L. & Hall, J. L. A Biochemical and Cytochemical Study of Peroxidase Activity in Roots of Pisum sativum II.Distribution of enzymes in relation to root development. *J. Exp. Bot.* **29**, 983-991 (1978).

7 He, J., Ji, Z.-x., Wang, Q.-z., Liu, C.-f. & Zhou, Y.-b. Effect of Cu and Pb pollution on the growth and antionxidant enzyme activity of Suaeda heteroptera. *Ecol. Eng.* **87**, 102-109, doi:10.1016/j.ecoleng.2015.11.004 (2016).

8 Aebi, H. Catalase in vitro. *Methods Enzymol.* **105**, 121-126 (1984).

9 Singh, A. & Prasad, S. M. A lucrative technique to reduce Ni toxicity in Raphanus sativus plant by phosphate amendment: Special reference to plant metabolism. *Ecotoxicol. Environ. Saf.* **119**, 81-89, doi:10.1016/j.ecoenv.2015.04.025 (2015).

10 Beauchamp, C. & Fridovich, I. Superoxide dismutase: improved assays and an assay applicable to acrylamide gels. *Anal. Biochem.* **44**, 276-287 (1971).

11 Islam, F. *et al.* Influence of Pseudomonas aeruginosa as PGPR on oxidative stress tolerance in wheat under Zn stress. *Ecotoxicol. Environ. Saf.* **104**, 285-293, doi:10.1016/j.ecoenv.2014.03.008 (2014).

12 Dhindsa, R. S. & Matowe, W. Drought Tolerance in Two Mosses: Correlated with Enzymatic Defence Against Lipid Peroxidation. *J. Exp. Bot.* **32**, 79-91 (1981).

13 Beiyuan, J. *et al.* Integrating EDDS-enhanced washing with low-cost stabilization of metal-contaminated soil from an e-waste recycling site. *Chemosphere* **159**, 426-432, doi:10.1016/j.chemosphere.2016.06.030 (2016).

14 Beiyuan, J. *et al.* Chelant-enhanced washing of CCA-contaminated soil: Coupled with selective dissolution or soil stabilization. *Sci. Total. Environ.* **612**, 1463-1472, doi:10.1016/j.scitotenv.2017.09.015 (2018).

15 Huang, B. *et al.* Distribution characteristics of heavy metal(loid)s in aggregates of different size fractions along contaminated paddy soil profile. *Environ. Sci. Poll. Res.* **24**, 23939-23952, doi:10.1007/s11356-017-0012-4 (2017).

16 Marrugo-Negrete, J., Pinedo-Hernández, J. & Díez, S. Assessment of heavy metal pollution, spatial distribution and origin in agricultural soils along the Sinú River Basin, Colombia. *Environ. Res.* **154**, 380-388 (2017).
